# Preoperative Decreased Hounsfield Unit Values of Cervical Vertebrae and the Relative Cross-Sectional Area of Flexion/Extension Paraspinal Muscles Are Novel Risk Factors for the Loss of Cervical Lordosis after Open-Door Laminoplasty

**DOI:** 10.3390/jcm12062119

**Published:** 2023-03-08

**Authors:** Wenjun Hu, Shaoguang Li, Huihong Shi, Yong Li, Jincheng Qiu, Jinlang Zhou, Dongsheng Huang, Yan Peng, Wenjie Gao, Anjing Liang

**Affiliations:** 1 Department of Orthopedics, Sun Yat-Sen Memorial Hospital, Yingfeng Road No. 33, Guangzhou 510130, China; 2 Department of Radiology, Sun Yat-Sen Memorial Hospital, Yingfeng Road No. 33, Guangzhou 510120, China

**Keywords:** open-door laminoplasty, loss of cervical lordosis, cervical alignment, cervical bone mineral density, HU values, asymmetry of paraspinal muscles, asymmetry of ranges of motion

## Abstract

Open-door laminoplasty is widely used for patients with cervical spondylotic myelopathy (CSM). However, the loss of cervical lordosis (LCL) seems to be unavoidable in the long-term follow-up after surgery, which may affect the clinical outcomes. The risk factors for this complication are still unclear. In this study, patients who underwent open-door laminoplasty between April 2016 and June 2021 were enrolled. Cervical X-rays were obtained to measure the C2–7 Cobb angle, C2–7 sagittal vertical axis (SVA), T1 slope (T1S) and ranges of motion (ROM). Cervical computed tomography (CT) scans and magnetic resonance imaging (MRI) were collected to evaluate the cervical Hounsfield unit values (HU) and the relative cross-sectional area (RCSA) of paraspinal muscles, respectively. A total of 42 patients were included and the average follow-up period was 24.9 months. Among the patients, 24 cases (57.1%) had a LCL of more than 5° at a 1-year follow-up and were labeled as members of the LCL group. The follow-up JOA scores were significantly lower in the LCL group (13.9 ± 0.6 vs. 14.4 ± 0.8, *p* = 0.021) and the mean JOA recovery rate was negatively correlated with LCL (*r* = −0.409, *p* = 0.007). In addition, LCL was positively correlated to the preoperative T1S, flexion ROM, flexion/extension ROM and the RCSA of flexion/extension muscles, while it was negatively correlated to extension ROM and the HU value of cervical vertebrae. Furthermore, multiple linear regression showed that preoperative T1S, mean HU value of cervical vertebrae, flexion/extension ROM and the flexion/extension RCSA were independent risk factors for LCL. Spine surgeons should consider these parameters before performing open-door laminoplasty.

## 1. Introduction

Cervical open-door laminoplasty has been widely used for patients with cervical spondylotic myelopathy (CSM) since its invention by Kiyoshi Hirabayashi in the 1970s [1,2]. Compared with cervical laminectomy, open-door laminoplasty can preserve the posterior structure and maintain cervical motion to some extent. However, the loss of cervical lordosis (LCL) or kyphotic deformity can still be observed in the long-term follow-up after surgery [3,4,5]. Lee et al. showed that postoperative LCL occurred in 80% of patients within a 1-year follow-up [6]. The cervical sagittal imbalance might affect the clinical outcomes. Xus et al. reported that the postoperative Japanese Orthopaedic Association scores (JOA scores) and JOA recovery rates were related to LCL after surgery [7]. It was also reported that patients with cervical kyphosis after laminoplasty had a worse prognosis [8].

Previous studies have investigated risk factors of LCL. Regarding parameters of preoperative cervical alignment, high T1S has been proven to be a risk factor for LCL [9,10,11]. Other studies showed that cervical ranges of motion (ROM) were also related to LCL [12,13]. Several studies found that a smaller relative cross-sectional area (RCSA) of the deep extensor muscles may lead to LCL [10,14]. However, whether the flexor muscles and bone quality of the cervical vertebrae affected the LCL has rarely been investigated.

Therefore, the risk factors of LCL are still controversial and require more comprehensive investigation. The purpose of the present study is to investigate preoperative parameters including cervical alignment, cervical bone quality and paraspinal muscles for predicting LCL after open-door laminoplasty.

## 2. Materials and Methods

### 2.1. Patients

This study is a retrospective study. A total of 56 patients who underwent open-door laminoplasty in our institution from April 2016 to June 2021 were enrolled. The exclusion criteria were as follows: (1) diagnoses of tumors, fractures, infections or spine deformity; (2) previous history of spine surgery; (3) follow-up of less than 1 year; and (4) lack of radiographic imaging or poor image quality. Two cases were excluded for diagnoses cervical tumors and four cases for a revision surgery. In total, 50 cases met the inclusion criteria. During the follow-up, 3 cases were excluded for lack of image data or poor quality of radiographs and 5 cases did not return a visit for personal reasons. Finally, 42 cases completed the follow-up. All cases were treated by the same medical group and received homogenous treatment, and they were all required to avoid strenuous exercise and trauma after surgery, while no other changes to the patients’ lifestyle were required. All patients received short-term NSAID painkillers to relieve neck pain from the incision after surgery, but none of them received any specialized physiotherapy after surgery and during follow-up.

### 2.2. Radiographic Assessment 

All patients underwent standard cervical spine X-ray (lateral, flexion and extension radiographs) preoperatively and lateral radiographs postoperatively at the last follow-up. CT scans and MRIs of the cervical spine were also obtained within 1 month before surgery. In the first year, postoperative patients were required to return to visit at 3, 6, 9 and 12 months after surgery, every 6 months in the second year and once every year after that. 

**Figure 1:**
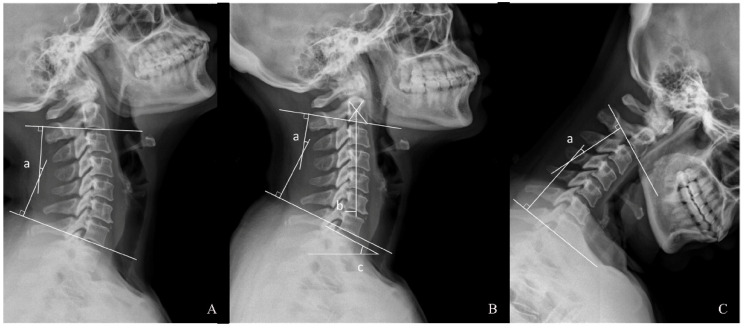
Radiographic measurements: (**A**) extension, (**B**) cervical lateral and (**C**) flexion radiographs, a. C2–7 Cobb angle, b. C2–7 sagittal vertical axis (SVA) and c. T1 Slope (T1S).

Cervical alignment parameters were measured as follows (Figure 1): cervical lordosis (CL), represented by C2–7 Cobb angle, was defined as the angle formed by the parallel lines of inferior endplates of C2 and C7. LCL was defined as a decrease in CL greater than 5° at the last follow-up [7]. C2–7 SVA was defined as the horizontal distance between the vertical line from the center of the C2 body and the posterior superior aspect of C7. T1S was defined as the angle between parallel lines of superior endplates of T1 and a horizontal line. The fROM was defined as the difference in C2–7 angles in the neutral and flexion positions while the eROM was defined as the difference in C2–7 angles in extension positions and neutral positions. The ROM was defined as the difference in C2–7 angles in extension positions and flexion positions. The ratio of fROM/eROM was calculated to represent the asymmetry of cervical mobility.

**Figure 2:**
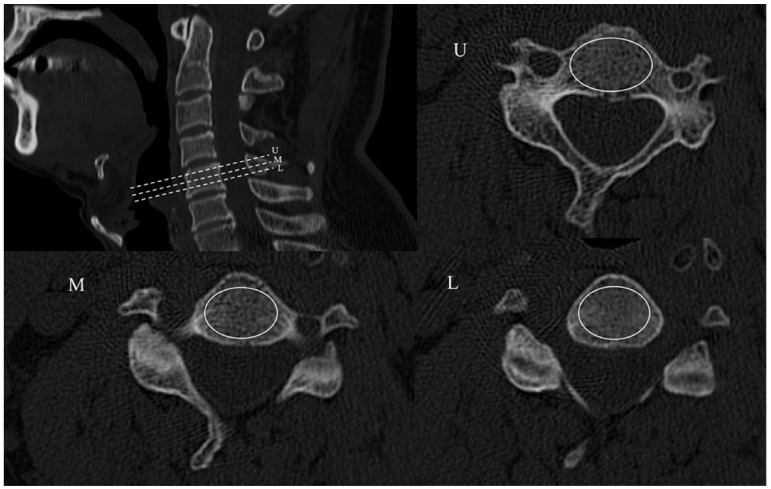
HU value measurements of cervical body on three slices: U. 2 mm below the superior endplate; M. in the middle of the vertebral body; L. 2 mm above the inferior endplate. The white oval represents the region of interest of the vertebral body on the axis plans.

The HU values of cervical vertebrae were measured through the hospital picture and archiving system (PACS) by a standard method published by Schreiber et al. [15]. We performed a 3D reconstruction of the CT film and chose three slices on the axis plane: in the middle of the vertebral body, 2 mm below the superior endplate and 2 mm above the inferior endplate. Then, we drew an oval region of interest (ROI) of the medullary space as large as possible (excluding the cortical margins to avoid volume averaging) on each slice to calculate the HU value (Figure 2). The HU value of each vertebral body was defined as the average HU value of three represented slices and the HU value of cervical spine was defined as the mean value of C2–C7.

**Figure 3:**
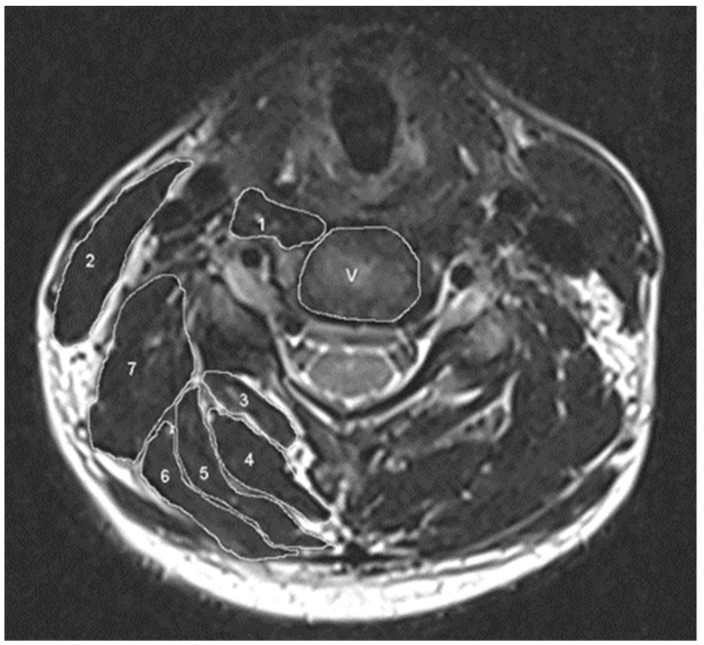
Measurement of the cross-sectional area (CSA) of the paraspinal muscles and vertebral body in C5/6 level by ImageJ software. The following paraspinal muscles were included: 1. longus colli and longus capitis; 2. sternocleidomastoid; 3. multifidus; 4. semispinalis cervicis; 5. semispinalis capitis; 6. splenius cervicis and splenius capitis; 7. levator scapulae and V. vertebral body.

ImageJ Software (version 1.52α, National Institutes of Health, Bethesda, MD, USA) was used to measure the cross-sectional area (CSA) of the paraspinal muscles. All data were collected on the level of C5/6, because previous research indicated that the muscles on these levels had the strongest correlation to the cervical paraspinal muscles and also because the surgical segment in all cases contains C4–C6. As shown in Figure 3, paraspinal muscles could be divided into flexion muscles and extension muscles. Flexion muscles included longus colli and longus capitis (LCo + LCa) and sternocleidomastoid (SCM). Extension muscles included multifidus (Mult), semispinalis cervicis (SeCe), semispinalis capitis (SeCa), splenius cervicis and splenius capitis (SpCe + SpCa) and levator scapulae (LSc). The CSAs of the C5 vertebral body were also measured as a reference point. The CSAs of muscles were manually outlined around the outer fascia and the software could calculate the area automatically [16,17]. The ratio of paraspinal muscle/vertebral body was calculated as the relative cross-sectional area and the flexion muscles/extension muscles ratio’s CSA was calculated to represent the asymmetry of cervical paraspinal muscles. 

All radiographic parameters were measured by two independent experienced observers and the average values were taken into consideration.

### 2.3. Clinical Function Assessment

Japanese Orthopaedic Association scores (JOA scores) were used to assess the cervical neurological function preoperatively and at the last follow-up. This self-rating scale includes 4 aspects: upper-limb function, lower-limb function, sensory function and bladder function. The score ranges from 0 (worst) to 17 (normal). The JOA recovery rate was calculated by the following formula: (postoperative JOA score-preoperative JOA score)/(17 − preoperative JOA score) × 100% [18].

### 2.4. Statistical Analysis

We used an independent sample *t*-test and a chi-square test to compare variables between groups and a paired sample *t*-test for the preoperative radiographic parameters and in the last follow-up. Pearson’s correlation analysis and Spearman correlation analysis were used to assess correlations between parameters and LCL. Multiple linear regression and the receiver operating characteristic curve (ROC) were used to investigate the risk factors for predicting LCL. Intra- and inter-rater reliability were assessed using the intraclass correlation coefficient (ICC) and ICC values greater than 0.8 was considered good. All data were analyzed via SPSS and a significant difference was defined as *p* < 0.05.

## 3. Results

A total of 42 (28 male and 14 female) patients were included in the present study. All the demographic data and clinical outcomes are shown in Table 1. The average age was 56.8 ± 8.4 years and the average BMI was 25.7 ± 2.3 kg/m^2^. The mean follow-up period was 24.9 ± 21.7 months. For the operation levels, 26 cases received laminoplasty at level C3-6 and the other 16 cases involved laminoplasty at C2 or C7. Improvements in JOA scores were observed in all patients at the last follow-up with an average recovery rate of 37.8 ± 14.7%. The mean C2–7 Cobb angle was 18.0 ± 9.7° preoperatively, and was 12.9 ± 10.8° at the last follow-up, with an average decrease of 5.1°. An increase in C2–7 SVA and decrease in T1S was also observed after surgery, which indicated that not only had the cervical lordosis decreased, but also that the whole cervical sagittal imbalance would appear after open-door laminoplasty. Other parameters of cervical alignment are also shown in Table 1.

As presented in Table 2 and Table 3, all parameters were compared between the LCL and NLCL groups. Baseline data including age, gender, BMI and whether the operation level involved C2/C7 or not were not significantly different between the two groups. Regarding the cervical alignment, preoperative C2–7 lordosis did not differ between the two groups while the LCL group had significantly smaller C2–7 Cobb angles at the last follow-up with a lower JOA score. For the preoperative parameters, higher T1S, larger fROM, smaller eROM and greater fROM/eROM were observed in the LCL group. As for the comparison of paraspinal muscles, the RCSA of LCo + Lca, total flexion muscles and flexor/extensor muscles were significantly larger in the LCL group. Additionally, the HU values of the C2–7 vertebral body respectively and the average values were significantly lower in the LCL group.

The results of correlation analysis between preoperative parameters and LCL are shown in Table 4. The JOA mean recovery rate was negatively correlated with the LCL (*r* = −0.430, *p* = 0.005). The preoperative T1S (*r* = 0.437, *p* = 0.004), fROM (*r* = 0.485, *p* = 0.001) and fROM/eROM (*r* = 0.545, *p* < 0.001) values were positively correlated with LCL while the eROM (*r* = −0.386, *p* < 0.011) showed a significant negative correlation. The average HU values of the C2–7 vertebral body showed a significant negative correlation with LCL (*r* = −0.352, *p* = 0.022). Regarding the RCSA of paraspinal muscles, the RCSA of LCo + Lca (*r* = 0.317, *p* = 0.041) and the RCSA of flexion/extension muscles (*r* = 0.421, *p* = 0.006) were positively correlated with LCL. Above all parameters, the fROM/eROM (*r* = 0.545) showed the strongest correlation with LCL.

**Figure 4:**
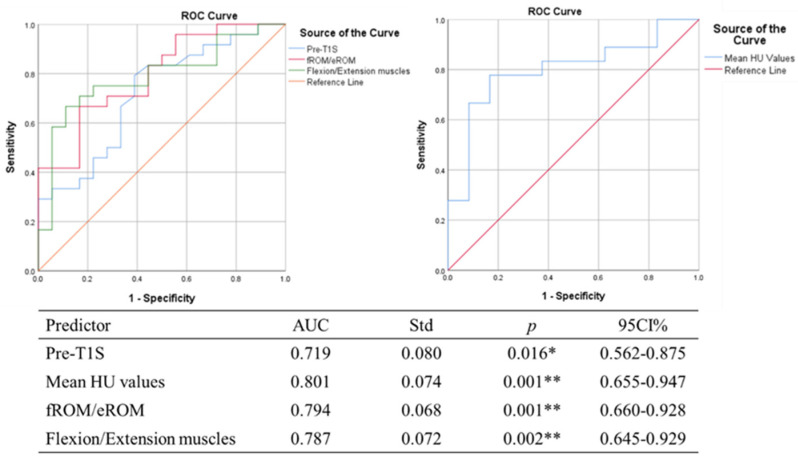
Receiver operating characteristic (ROC) curve for prediction of LCL. Pre, Preoperative; T1S, T1 slope; HU, Hounsfield Units; fROM, ranges of flexion motion; eROM, ranges of extension motion. * *p* < 0.05, statistical significance. ** *p* < 0.01, statistical significance.

Multiple linear regression showed that preoperative T1S, mean cervical HU value, fROM/eROM and flexion/extension RCSA were independent risk factors for LCL, as shown in Table 5. The ROC curve showed that the corresponding AUC values were 0.719, 0.801, 0.794 and 0.787, respectively, as shown in Figure 4.

## 4. Discussion

Open-door laminoplasty is a common procedure for multiple-level cervical spondylotic myelopathy. Indirect decompression is achieved with a posterior shift of the spinal cord at the expense of the spinal canal. However, the loss of cervical lordosis (LCL) seems to be inevitable after surgery, which may lead to decompression failure and poor clinical outcomes [5,6,7,8]. A decrease in C2–7 Cobb angle was observed in 32 (76.2%) patients in the 1-year follow-up and, among them, 24 cases (57.1%) had a loss of cervical lordosis of more than 5°. The mean C2–7 Cobb angle was reduced from 18.0° preoperatively to 12.9°. 

Several risk factors for LCL after laminoplasty have been investigated in previous studies. It was reported by Zhang [9] and Kim [10] that a higher preoperative T1S was an independent risk factor of LCL. Lee et al. found that patients with preoperative TIS > 29° faced a higher risk for a decrease in lordosis of more than 5° [19]. In the present study, patients in the LCL group had a higher preoperative T1S and this was moderately related to a decrease in C2–7 Cobb angle. Multiple linear regression showed that it was an independent risk factor for LCL. Patients with high T1S may also have large cervical lordosis as compensation to maintain a horizontal gaze and a balanced sagittal alignment, which requires extra work provided by the cervical paraspinal muscles. The primary degeneration or asymmetry of paraspinal muscles or damage from operation might lead to weakness of paraspinal muscles after surgery, resulting in LCL.

The roles of cervical range of motion and extension muscles in the occurrence of postoperative LCL were also recently noticed. Previous studies focused more on the role of extension range of motion (eROM). A study by Lee showed that a smaller extension range of motion (eROM) was an independent predictor of LCL and the cutoff value was 14° [12] and another study demonstrated that eROM was related to LCL after laminoplasty [20]. However, recent studies have suggested that the flexion range of motion (fROM) should not be ignored. The fROM could be used to distinguish the LCL group from the NLCL group and a greater fROM was related to LCL [13]. A recent study by Masashi showed that a composite indicator, which was the gap between fROM and eROM, was a highly useful indicator for LCL [21]. Smaller fROM or ROM may be caused by degenerative structures including ossification of the posterior longitudinal ligament, osteophytes or degenerative intervertebral disc, which limits the cervical alignment change from lordosis to kyphosis. Conversely, patients with larger fROM or eROM may have more potential to change cervical lordosis. In the present study, the fROM, eROM and the ratio of fROM/eROM, which represents the asymmetry of cervical mobility, showed correction with LCL. Additionally, fROM/eROM was investigated to be an independent risk factor for LCL by multiple linear regression analysis.

The paraspinal muscles play an important role in maintaining cervical lordosis and balance [22,23]. The semispinalis cervicis was found to be the most important muscle in maintaining cervical lordosis [24], and preoperative CSA of the semispinalis cervicis was related to LCL [14]. In addition, the CSA of deep extensor muscles was smaller in the LCL group according to a study conducted by Kim [10]. A recent study found that the asymmetry of cervical extension muscles at the C6 level was associated with cervical sagittal imbalance after laminoplasty [25]. However, little attention has been paid to the anterior flexion muscles. We hypothesized that the cervical flexion muscles also played an important role in maintaining spinal alignment balance and that the asymmetry of paraspinal muscles may be related to LCL. In the present study, the RCSA of longus colli and longus capitis and the RCSA of total flexion muscles were significantly larger in the LCL group. Correlation analysis showed that the flexion/extension muscles ratio’s RCSA was positively related to LCL. Additionally, multiple regression analysis confirmed that it was a risk factor for LCL. As mentioned above, extension muscles provide the main work in maintaining cervical lordosis. The damage from surgery caused degeneration and weakness of the flexion muscles, which may further aggravate the imbalance of the flexion and extension muscles, resulting in LCL. A previous retrospective study mentioned that for patients who underwent expansive laminoplasty, cases in a postoperative No Pain Group (VAS < 3) had stronger neck muscle strength and higher muscle strength recovery rate at follow-up compared to a postoperative Pain Group (VAS ≥ 3) [26]. Proper neck muscle exercises may help to maintain cervical alignment and relieve neck pain.

Whether the cervical bone quality was related to LCL after open-door laminoplasty has scarcely been investigated. Previous studies have shown that the HU values of the vertebral body in CT scans was reliable for assessing bone mineral density (BMD) [15,27]. In the present study, the HU values of each cervical of C2–7 and the average value were all significantly lower in the LCL group. In addition, the average cervical HU value showed negative correlation with the LCL. Multiple linear regression showed that the HU value of C2–7 was another independent risk factor for LCL. Several studies demonstrated the relationship between cervical sagittal alignment and cervical HU values. Lovecchio et al. showed that the cervical HU values were positively correlated with cervical kyphosis [28]. Another study investigated whether vertebral wedging was more likely to occur in patients with osteoporosis and aging people [29]. Loss of horizontal trabeculae in the vertebrae increases the risk of minor anterior vertebral body fracture and wedge deformity [30]. Additionally, a negative effect on BMD might occur in the early phase after cervical or lumbar surgery [31,32]. Thus, patients with low cervical HU values may have higher risk of vertebral wedge change, which results in unstable alignment and a decrease in cervical lordosis. 

This study had some limitations. Firstly, it is a retrospective study from a single center. A prospective study with a larger sample size is needed to provide a higher level of evidence. Secondly, only a few cases obtained a whole-spine X-ray so we could not investigate the effect from thoracic and lumbar vertebrae on cervical alignments. Thirdly, because all of the radiographic parameters, the ROI of HU and RCSA of muscles were measured manually, it was difficult to recognize the boundary of vertebral bodies and muscles in some cases, which may cause errors.

## 5. Conclusions

The loss of cervical lordosis is a common complication after open-door laminoplasty and results in poor prognosis. Preoperative T1S, C2–7 HU values and the asymmetry of fROM/eROM and flexion/extension paraspinal muscles were risk factors for LCL. Spine physicians should evaluate these parameters before performing open-door laminoplasty and pay attention to pre–postoperative cervical muscle-strengthening exercises.

## Figures and Tables

**Table 1:** Basal characteristics and clinical outcomes of the study population.

Variable	Descriptive Statistics	*p*
Age (year)	56.8 ± 8.4	
Sex		
Male	28 (66.7%)	
Female	14 (33.3%)	
BMI (kg/m^2^)	25.7 ± 2.29	
Follow-up period (month)	24.9 ± 21.7	
Operation level		
C3–6	26 (61.9%)	
Involve C2/C7	16 (38.1%)	
JOA score		
Preoperative	12.2 ± 1.2	
Follow-up	14.1 ± 0.72	<0.01 **
JOA mean recovery rate	37.8 ± 14.7%	
C2–7 Cobb (°)		
Preoperative	18.0 ± 9.7	
Follow-up	12.9 ± 10.8	<0.01 **
C2–7 SVA (mm)		
Preoperative	12.9 ± 6.5	
Follow-up	17.2 ± 11.9	0.027 *
T1S (°)		
Preoperative	24.9 ± 6.4	
Follow-up	22.7 ± 7.8	0.024 *
fROM (°)	33.5 ± 9.8	
eROM (°)	8.5 ± 5.0	
ROM (°)	42.1 ± 9.7	
fROM/eROM	9.8 ± 23.9	
eROM/fROM	0.21 ± 0.13	

BMI, body mass index; CSM, cervical spondylotic myelopathy; JOA score, Japanese Orthopedic Association Score; SVA, sagittal vertical axis; T1S, T1 slope; ROM, ranges of motion; fROM, ranges of flexion motion; eROM, ranges of extension motion. * *p* < 0.05, statistical significance. ** *p* < 0.01, statistical significance.

**Table 2:** Comparation of basal characteristics and clinical outcomes between LCL and NLCL groups.

Variable		LCL	NLCL	*p*
Age		58.1 ± 8.0	55.2 ± 8.9	0.264
Sex (M/F)		18/6	10/8	0.186
BMI (kg/m^2^)		25.74	25.90	0.268
C2/C7 Involved (Yes/No)		8/16	10/8	0.211
Follow-up period (month)		23.7 ± 20.3	26.7 ± 23.9	0.664
JOA score	Preoperative	12.2 ± 1.0	12.2 ± 1.5	0.887
	Follow-up	13.9 ± 0.6	14.4 ± 0.8	0.021 *
Mean JOA recovery rate		34.1 ± 11.6	42.9 ± 17.1	0.053

* *p* < 0.05, statistical significance.

**Table 3:** Comparation of radiographic parameters between LCL and NLCL groups.

Variable		LCL	NLCL	*p*
C2–7 Cobb (°)	Preoperative	19.49 ± 8.20	15.98 ± 11.41	0.253
	Follow-up	9.20 ± 9.33	17.88 ± 10.88	<0.01 **
T1S (°)	Preoperative	27.46 ± 6.39	22.42 ± 6.48	0.016 *
	Follow-up	24.23 ± 8.42	21.25 ± 7.12	0.233
C2–7 SVA (mm)	Preoperative	11.91 ± 5.70	14.28 ± 7.33	0.244
	Follow-up	21.73 ± 11.88	11.10 ± 9.11	<0.01 **
fROM (°)	Preoperative	38.63 ± 7.54	26.71 ± 8.37	<0.01 **
eROM (°)	Preoperative	7.11 ± 4.62	10.43 ± 4.95	0.031 *
ROM (°)	Preoperative	45.7 ± 7.8	37.14 ± 9.97	<0.01 **
fROM/eROM	Preoperative	14.67 ± 30.99	3.34 ± 2.18	<0.01 **
Muscles RCSA				
LCo + LCa	Preoperative	0.47 ± 0.12	0.38 ± 0.10	0.019 *
SCM	Preoperative	1.59 ± 0.40	1.39 ± 0.30	0.087
Mult	Preoperative	0.39 ± 0.15	0.46 ± 0.11	0.139
SeCe	Preoperative	0.49 ± 0.14	0.56 ± 0.17	0.144
SeCa	Preoperative	0.67 ± 0.24	0.77 ± 0.22	0.157
SpCe + SpCa	Preoperative	0.90 ± 0.21	0.84 ± 0.20	0.295
LSc	Preoperative	1.17 ± 0.22	1.19 ± 0.31	0.790
Flexion muscles	Preoperative	2.06 ± 0.48	1.77 ± 0.38	0.046 *
Extension muscles	Preoperative	3.62 ± 0.72	3.81 ± 0.90	0.441
Flexion/Extension muscles	Preoperative	0.57 ± 0.11	0.47 ± 0.08	<0.01 **
HU values				
C2	Preoperative	354.75 ± 86.31	429.22 ± 81.02	0.007 **
C3	Preoperative	310.29 ± 68.60	414.83 ± 83.33	<0.01 **
C4	Preoperative	328.13 ± 64.55	428.44 ± 102.03	<0.01 **
C5	Preoperative	317.04 ± 68.53	408.94 ± 97.88	<0.01 **
C6	Preoperative	260.67 ± 58.20	335.06 ± 87.22	<0.01 **
C7	Preoperative	244.58 ± 57.43	302.17 ± 65.23	<0.01 **
Mean HU values	Preoperative	302.75 ± 64.70	386.61 ± 81.89	<0.01 **

RCSA = relative cross-sectional area; LCo + LCa, longus colli and longus capitis; SCM, sternocleidomastoid; Mult, multifidus; SeCe, semispinalis cervicis; SeCa, semispinalis capitis; SpCe + SpCa, splenius cervicis and splenius capitis; LSc, levator scapulae; HU, Hounsfield Units. * *p* < 0.05, statistical significance. ** *p* < 0.01, statistical significance.

**Table 4:** Correlation between various factors and LCL.

		Age	BMI	JOA Mean Recovery Rate	Pre- C2–7 Cobb	Pre-T1S	Pre- C2–7 SVA	fROM	eROM	ROM	fROM/eROM	LCo + LCa RCSA	SCMRCSA	Mult RCSA	SeCe RCSA	SeCa RCSA	SpCe + SpCa RCSA	LSc RCSA	Flexion Muscles RCSA	Extension Muscles RCSA	Flexion/Extension Muscles RCSA	HU Values
LCL	*r*	−0.088	0.037	−0.430	−0.260	0.437	0.034	0.485	−0.386	0.293	0.545	0.317	0.145	−0.264	−0.279	−0.301	0.079	−0.066	0.182	−0.158	0.421	−0.352
	*p*	0.869	0.814	0.005 **	0.096	0.004 **	0.833	0.001 **	0.011 **	0.060	0.001 **	0.041 *	0.360	0.091	0.074	0.053	0.617	0.679	0.248	0.038	0.006 **	0.022 *

Pre, preoperative. * *p* < 0.05, statistical significance. ** *p* < 0.01, statistical significance.

**Table 5:** Association between various factors and LCL in multiple linear regression analysis.

	Unstandardized Coefficients		Standardized Coefficients		*p* Value
	*Β*	SE	*β*	*t*	
Constant	−9.822	8.622		−1.139	0.262
Pre-T1S	0.422	0.145	0.367	2.910	<0.01 **
Mean HU values	−0.029	0.041	−0.305	2.666	0.018 *
fROM/eROM	0.111	0.012	0.336	−2.479	0.011 *
Flexion/Extension muscles	24.276	8.881	0.336	2.733	0.010 *

* *p* < 0.05, statistical significance. ** *p* < 0.01, statistical significance.

## Data Availability

The datasets generated and analyzed during the current study are not publicly available due to the fact that they constitute an excerpt of research in progress but are available from the corresponding author on reasonable request.

## References

[1] Hirabayashi K., Watanabe K., Wakano K., Suzuki N., Satomi K., Ishii Y. (1983). Expansive open-door laminoplasty for cervical spinal stenotic myelopathy. Spine.

[2] Hirabayashi K., Watanabe K. (2019). A Review of My Invention of Expansive Laminoplasty. Neurospine.

[3] Aita I., Wadano Y., Yabuki T. (2000). Curvature and range of motion of the cervical spine after laminaplasty. J. Bone Jt. Surg. Am..

[4] Ogawa Y., Toyama Y., Chiba K., Matsumoto M., Nakamura M., Takaishi H., Hirabayashi H., Hirabayashi K. (2004). Long-term results of expansive open-door laminoplasty for ossification of the posterior longitudinal ligament of the cervical spine. J. Neurosurg. Spine.

[5] Suk K.S., Kim K.T., Lee J.H., Lee S.H., Lim Y.J., Kim J.S. (2007). Sagittal alignment of the cervical spine after the laminoplasty. Spine.

[6] Lee J.S., Son D.W., Lee S.H., Kim D.H., Lee S.W., Song G.S. (2017). The Predictable Factors of the Postoperative Kyphotic Change of Sagittal Alignment of the Cervical Spine after the Laminoplasty. J. Korean Neurosurg. Soc..

[7] Xu C., Zhang Y., Dong M., Wu H., Yu W., Tian Y., Cao P., Chen H., Wang X., Shen X. (2020). The relationship between preoperative cervical sagittal balance and clinical outcome of laminoplasty treated cervical ossification of the posterior longitudinal ligament patients. Spine J..

[8] Koda M., Mochizuki M., Konishi H., Aiba A., Kadota R., Inada T., Kamiya K., Ota M., Maki S., Takahashi K. (2016). Comparison of clinical outcomes between laminoplasty, posterior decompression with instrumented fusion, and anterior decompression with fusion for K-line (-) cervical ossification of the posterior longitudinal ligament. Eur. Spine J..

[9] Zhang J.T., Li J.Q., Niu R.J., Liu Z., Tong T., Shen Y. (2017). Predictors of cervical lordosis loss after laminoplasty in patients with cervical spondylotic myelopathy. Eur. Spine J..

[10] Kim K.R., Lee C.K., Park J.Y., Kim I.S. (2020). Preoperative Parameters for Predicting the Loss of Lordosis After Cervical Laminoplasty. Spine.

[11] Kim B., Yoon D.H., Ha Y., Yi S., Shin D.A., Lee C.K., Lee N., Kim K.N. (2016). Relationship between T1 slope and loss of lordosis after laminoplasty in patients with cervical ossification of the posterior longitudinal ligament. Spine J..

[12] Lee S.H., Son D.W., Lee J.S., Sung S.K., Lee S.W., Song G.S. (2019). Does Extension Dysfunction Affect Postoperative Loss of Cervical Lordosis in Patients Who Undergo Laminoplasty?. Spine.

[13] Fujishiro T., Nakano A., Yano T., Nakaya Y., Hayama S., Usami Y., Nozawa S., Baba I., Neo M. (2020). Significance of flexion range of motion as a risk factor for kyphotic change after cervical laminoplasty. J. Clin. Neurosci. Off. J. Neurosurg. Soc. Australas..

[14] Lee B.J., Park J.H., Jeon S.R., Rhim S.C., Roh S.W. (2018). Importance of the preoperative cross-sectional area of the semispinalis cervicis as a risk factor for loss of lordosis after laminoplasty in patients with cervical spondylotic myelopathy. Eur. Spine J..

[15] Schreiber J.J., Anderson P.A., Rosas H.G., Buchholz A.L., Au A.G. (2011). Hounsfield units for assessing bone mineral density and strength: A tool for osteoporosis management. J. Bone Jt. Surg. Am..

[16] Fortin M., Battié M.C. (2012). Quantitative paraspinal muscle measurements: Inter-software reliability and agreement using OsiriX and ImageJ. Phys. Ther..

[17] Huang Z., Bai Z., Yan J., Zhang Y., Li S., Yuan L., Huang D., Ye W. (2022). Association Between Muscle Morphology Changes, Cervical Spine Degeneration, and Clinical Features in Patients with Chronic Nonspecific Neck Pain: A Magnetic Resonance Imaging Analysis. World Neurosurg..

[18] Furlan J.C., Catharine Craven B. (2016). Psychometric analysis and critical appraisal of the original, revised, and modified versions of the Japanese Orthopaedic Association score in the assessment of patients with cervical spondylotic myelopathy. Neurosurg. Focus.

[19] Lee B.S., Walsh K.M., Lubelski D., Knusel K.D., Steinmetz M.P., Mroz T.E., Schlenk R.P., Kalfas I.H., Benzel E.C. (2018). The effect of C2-3 disc angle on postoperative adverse events in cervical spondylotic myelopathy. J. Neurosurg. Spine.

[20] Sharma R., Borkar S., Katiyar V., Goda R., Phalak M., Joseph L., Suri A., Chandra P.S., Kale S.S. (2020). Interplay of Dynamic Extension Reserve and T1 Slope in Determining the Loss of Cervical Lordosis Following Laminoplasty: A Novel Classification System. World Neurosurg..

[21] Fujishiro T., Hayama S., Obo T., Nakaya Y., Nakano A., Usami Y., Nozawa S., Baba I., Neo M. (2021). Gap between flexion and extension ranges of motion: A novel indicator to predict the loss of cervical lordosis after laminoplasty in patients with cervical spondylotic myelopathy. J. Neurosurg. Spine.

[22] Iizuka H., Nakajima T., Iizuka Y., Sorimachi Y., Ara T., Nishinome M., Takagishi K. (2007). Cervical malalignment after laminoplasty: Relationship to deep extensor musculature of the cervical spine and neurological outcome. J. Neurosurg. Spine.

[23] Iizuka H., Shimizu T., Tateno K., Toda N., Edakuni H., Shimada H., Takagishi K. (2001). Extensor musculature of the cervical spine after laminoplasty: Morphologic evaluation by coronal view of the magnetic resonance image. Spine.

[24] Vasavada A.N., Li S., Delp S.L. (1998). Influence of muscle morphometry and moment arms on the moment-generating capacity of human neck muscles. Spine.

[25] Lin S., Lin T., Wu Z., Chen G., Shangguan Z., Wang Z., Liu W. (2022). Does the asymmetry and extension function of the preoperative cervical paraspinal extensor predict postoperative cervical sagittal deformity in patients who undergo modified laminoplasty?. Spine J..

[26] Fujibayashi S., Neo M., Yoshida M., Miyata M., Takemoto M., Nakamura T. (2010). Neck muscle strength before and after cervical laminoplasty: Relation to axial symptoms. J. Spinal Disord. Tech..

[27] Colantonio D.F., Saxena S.K., Vanier A., Rodkey D., Tintle S., Wagner S.C. (2020). Cervical Spine Computed Tomography Hounsfield Units Accurately Predict Low Bone Mineral Density of the Femoral Neck. Clin. Spine Surg..

[28] Lovecchio F., Ang B., Louie P.K., Chaudary C., Shah S.P., Punyala A., Yao Y.C., Steinhaus M., McCarthy M.H., Huang R. (2022). Bone Density Distribution in the Cervical Spine. Glob. Spine J..

[29] Yang Z., Griffith J.F., Leung P.C., Lee R. (2009). Effect of osteoporosis on morphology and mobility of the lumbar spine. Spine.

[30] Landham P.R., Gilbert S.J., Baker-Rand H.L., Pollintine P., Robson Brown K.A., Adams M.A., Dolan P. (2015). Pathogenesis of Vertebral Anterior Wedge Deformity: A 2-Stage Process?. Spine.

[31] Salzmann S.N., Okano I., Miller C.O., Chiapparelli E., Reisener M.J., Amini D.A., Winter F., Shue J., Carrino J.A., Sama A.A. (2022). The cervical spine demonstrates less postoperative bone loss than the lumbar spine. J. Orthop. Res. Off. Publ. Orthop. Res. Soc..

[32] Demir Ö., Öksüz E., Deniz F.E., Demir O. (2017). Assessing the effects of lumbar posterior stabilization and fusion to vertebral bone density in stabilized and adjacent segments by using Hounsfield unit. J. Spine Surg..

